# Blind Deconvolution for Ultrasound Sequences Using a Noninverse Greedy Algorithm

**DOI:** 10.1155/2013/496067

**Published:** 2013-12-29

**Authors:** Liviu-Teodor Chira, Corneliu Rusu, Clovis Tauber, Jean-Marc Girault

**Affiliations:** ^1^Signal & Imaging Group, University François Rabelais of Tours, PRES Loire Valley University, UMR INSERM U930, 7 Avenue Marcel Dassault, 37200 Tours Cedex, France; ^2^Faculty of Electronics, Telecommunications and Information Theory, Technical University of Cluj-Napoca, Cluj-Napoca 400027, Romania

## Abstract

The blind deconvolution of ultrasound sequences in medical ultrasound technique is still a major problem despite the efforts made. This paper presents a blind noninverse deconvolution algorithm to eliminate the blurring effect, using the envelope of the acquired radio-frequency sequences and *a priori* Laplacian distribution for deconvolved signal. The algorithm is executed in two steps. Firstly, the point spread function is automatically estimated from the measured data. Secondly, the data are reconstructed in a nonblind way using proposed algorithm. The algorithm is a nonlinear blind deconvolution which works as a greedy algorithm. The results on simulated signals and real images are compared with different state of the art methods deconvolution. Our method shows good results for scatters detection, speckle noise suppression, and execution time.

## 1. Introduction

Medical ultrasound imaging is considered to be one of the edge technologies in noninvasive diagnose procedures. Despite its great advantages, as cost-benefit, accessibility, portability, and safety, it has a weak resolution. This is the result of the attenuations, refractions, nonlinearities, frequency selection, or probe properties [1]. As a result, important efforts were made in the direction of image quality improvement.

Signal processing methods offer a reasonable approach for resolution improvement. From this point of view the most important methods for reconstruction are superresolution and deconvolution. If superresolution methods seem to be impractical, the deconvolution ones are more practical [2]. Supperresolution is a complex problem because of the difficulties in aproximation of reconstruction operators (e.g., motion, degradation, and subsampling operators) and the use of multiple frames which puzzles also the implementation. This was conducted on the proposition of multiple deconvolution approaches for ultrasound imaging, like methods used in system identification or Bayesian statistics based ones [3]. From these algorithms, the methods based on Bayesian approach, especially maximum *a Posteriori* (MAP) seem to offer the most interesting results [4–10]. In these methods the point spread function (PSF) is estimated and then the information is reconstructed in a nonblind way using *a priori* information about tissue reflectivity function.

As the PSF estimation is an important problem that is complex, a lot of methods were advanced to propose an acceptable solution. Primary studies have considered a measured radio-frequency (RF) PSF [4, 11]. However, the use of only one RF PSF to deconvolve the entire image is not feasible due to the nonstationarity of the PSF along the RF line which results from the attenuations, reflections, refractions, and phase aberrations phenomena. A common solution is to estimate the PSF locally by supposing that it is a slow variant in time. This needs to divide the image in segments where one may consider that the PSF is constant and can be estimated for each segment.

Based on the local estimation of the PSF, a certain number of methods were proposed. An approach based on a 1D implementation was proposed to estimate the RF PSF using high order statistics [5].

Taxt et al. introduced a method of RF PSF estimation using the cepstrum and homomorphic deconvolution [6, 12–14]. In this approach it is considered that the PSF spectrum is a function smoother than the reflectivity function.

Another method of unidimensional RF PSF estimation from ultrasound sequences was proposed in [15]. This approach was developed for the cases when the interrogated tissues are composed by high reflectors superimposed by speckle noise, that is, the reflectivity function has Laplacian distribution. For that, it was proposed a complex homomorphic procedure, where for the elimination of the spectrum of reflectivity function a multilevel decomposition denoising technique was used [16]. This denoising technique was improved with an outlier resistant denoising procedure [17] and the phase was estimated using minimum phase assumption.

From the point of view of nonblind algorithms used for deconvolution, the proposed methods supposed that the reflectivity function has a Gaussian or a Laplacian probability of distribution function. For that it was frequently used the Wiener filter (or *l*_2_-norm regularization) [4–6, 11–14] or *l*_1_-norm regularization [7, 9]. If the Wiener filtering seems to smooth the information and to offer a resulted image with a small resolution improvement and speckle noise suppression, the methods based on Laplacian distribution showed a better improvement in terms of contrast and speckle noise reduction.

Other kinds of approaches were proposed in [8] or [10]. In [8] the authors proposed an expectation-maximization algorithm that solved the problem iteratively, by alternating between Wiener filtering and wavelet-based denoising. In [10], 2-steps deconvolution algorithm was proposed, where in first step the PSF is estimated using the Cepstrum technique and for deconvolution a two steps iterative shrinkage/thresholding (TwIST) is used.

However, all the previous methods suffer of difficulties in the phase approximation of the RF PSF in the algorithm robustness. To overcome these difficulties recent works were focused to extract the reflectivity function using the envelope of RF data [8–10, 18]. The most important part of algorithms based on RF envelope intends to extract the tissue reflectivity function using the idea of the inverse filtering. These methods imply matrix inversion which may produce singularities. For such reason, it was necessary to introduce regularization methods which ask for additional computation.

In this paper two major contributions are proposed. The first contribution concerns the combination of three main steps that are the envelope detection based on Hilbert transform, the PSF estimation based on a general homomorphic deconvolution approach, and the deconvolution algorithm based on greedy implementation. Knowing that each method taken alone is not novel, this combination of the above three steps constitutes a novelty. The second contribution concerns the application's field since it is the first time when it is used in ultrasound medical imaging.

Being a blind algorithm, it performs the reconstruction process automatically in two steps. Firstly, the PSF is estimated for each sequence composing the ultrasound image, and, secondly the reflectivity function is obtained using proposed algorithm with the *a priori* assumption that reflectivity function is a sparse signal; that is, it has a Laplacian probability of density function (PDF). The proposed approach is an iterative algorithm based on the matching pursuit [19] principle that avoids the difficult *inverse problem* in signal reconstruction [20]. Finally, to take into account that PSF can be variant with depth, note that the proposed method can be used on short time subsequences derived from the analyzed sequence at different depths.

In the following, the paper is organized as follows.  Section 2 describes the problem reconstruction for ultrasound imaging, Section 3 presents the proposed method, Section 4 shows the experimental results, and Section 5 provides several comments and concludes the current study.

## 2. Problem Formulation in Ultrasound Medical Images Restoration

In ultrasound imaging the obtained A-mode and B-mode images suppose the interaction between the acoustic beam, generated by the transducer and the scanned tissues. Usually, the phenomena are not linear but for computations simplicity the greatest part of the methods proposed in the literature suppose that the acquired signal is a quasi-linear combination between the reflectivity function and the RF pulse. This supposes that the ultrasound sequences are divided in segments and each segment is processed individually. For the sake of simplicity we reduce the analysis to singular segment. The mathematical formulation of the measured signal *y*(*n*) can be described as follows:

(1) $$\begin{array}{c} y\left(n\right)=h\left(n\right)⊗x\left(n\right)+u\left(n\right), \end{array}$$

where ⊗ is the convolution operator, *h*(*n*) is the PSF, *x*(*n*) is reflectivity function, *u*(*n*) is a Gaussian white noise, and *n* is the samples index. In the frequency domain, (1) can be written as

(2) $$\begin{array}{c} Y\left(\omega \right)=H\left(\omega \right)\cdot X\left(\omega \right)+U\left(\omega \right), \end{array}$$

where the upper case letters represent the Fourier Transform (FT) of the components from (1) and *ω* is the angular frequency.

As previously mentioned in Section 1, the purpose of the blind reconstruction methods is to obtain the true signal *x*(*n*) starting from the acquired signal *y*(*n*). A natural solution from (2) is to obtain *X*(*ω*) by inverting *H*(*ω*), which is the FT of the PSF. Equation (2) can be rewritten as

(3) $$\begin{array}{c} X\left(\omega \right)=\frac{1}{H\left(\omega \right)}Y\left(\omega \right)-\frac{1}{H\left(\omega \right)}U\left(\omega \right). \end{array}$$

The main problem is that the small values of *H*(*ω*) will amplify by its inversion the high frequencies and implicitly the noise. The most used solutions on this problem are the regularization, according to PDF of the reconstructed signals. In ultrasound imaging a part of the generated pulse is reflected when it finds an interface between two tissues with different physical properties. Therefore, we classically suppose that the reflectivity function has a Laplacian PDF [7, 9, 10, 15].

## 3. Envelope Based Blind Deconvolution

The acquired signal can be described like an amplitude modulated signal, where the carrier is the wave generated by the transducer and the information is located in its envelope. Based on this assumption, the proposed method starts from the acquired RF signals. From these signals, we extract the envelope and afterwards, this envelope is used in two steps blind deconvolution algorithm: in the first step, the PSF is extracted; then it is used in the greedy noninverse deconvolution algorithm. In the following sections we detail the methods implemented in this paper:

- hilbert transform for envelope detection;
- homomorphic deconvolution and soft-thresholding denoising for PSF estimation;
- noninverse greedy deconvolution.

### 3.1. Envelope Detection

The most popular methods for envelope detection are Hilbert transform or low-pass filtering to separate the useful information contained in envelope from the sinusoidal RF carrier wave. Since the design of low-pass filter may be critical due to the unclear signals spectrum specifications, in this work the Hilbert transform has been preferred. The envelope *y*(*n*) can be extracted by applying the absolute value operator at the analytic signal as follows:

(4) $$\begin{array}{c} y\left(n\right)=\left|y_{a}\left(n\right)\right|, \end{array}$$

where the *y*_*a*_(*n*) means the analytic signal, *y*(*n*) means the obtained envelope, and |·| is the absolute value operator. The analytic signal is generated using Hilbert transform as follows:

(5) $$\begin{array}{c} y_{a}\left(n\right)=y_{\text{RF}}\left(n\right)+jℋ\left\{y_{\text{RF}}\left(n\right)\right\}, \end{array}$$

where *y*_*a*_(*n*) is the analytic signal, *y*_RF_(*n*) is the original RF signal, and *ℋ*{*y*_RF_(*n*)} is the Hilbert transform of *y*_RF_(*n*).

### 3.2. Point Spread Function Estimation

In ultrasound imaging it was widely assumed in many works that the PSF is a much smoother function than the tissue reflectivity function and that two composing signals of the measured signal spectrum can be separated using homomorphic deconvolution [21] and the denoising procedure as in [15]. The greatest advantage of this kind of homomorphic filters is that they may accept as input a signal composed of two components and return a signal with one of them removed.

The proposed estimation is a three steps algorithm; in the first step we assume that the noise level in (2) is quite small and we may ignore it. In this case it can be rewitten as follows [21]:

(6) $$\begin{array}{c} \ln Y\left(\omega \right)=\ln H\left(\omega \right)+\ln X\left(\omega \right), \end{array}$$

where ln⁡ is the natural logarithm. In this way the output signal is split into two parts: a part which comes from PSF and another one, which occurs from the input signal.

This linear transformation helps us to make a distinction between the signals, under the above presented assumptions that PSF is a smoother function. Thus, the wave separation problem could be changed in a denoising one. This is in the second step of the homomorphic deconvolution. The main idea of this technique is the use of a denoising method in the frequency domain by applying a wavelet soft thresholding and an outlier resistant denoising algorithm. The threshold was calculated as follows [22]:

(7) $$\begin{array}{c} T=\sigma \sqrt{2\ln N}, \end{array}$$

where *N* is the length of the array and *σ* is the noise variance. The *σ* parameter is automatically estimated by *σ* = *M*_*x*_/0.6745, where *M*_*x*_ was the median absolute value of the finest decomposition level.

Having obtained ln⁡*H*(*ω*), the final step of the homomorphic deconvolution is to get the PSF *h*(*n*) by using the Inverse Fourier Transform (IFT) of the logarithm spectrum of the PSF, as follows:

(8) $$\begin{array}{c} h\left(n\right)=\text{IFT}\left\{\exp\left[\ln\left(H\left(\omega \right)\right)\right]\right\}. \end{array}$$

In our implementation, the Fourier Transform was evaluated using Discrete Fourier Transform (DFT).

### 3.3. Greedy Deconvolution Algorithm

This section describe the proposed algorithm for reflectivity function recovery. It is a greedy algorithm analogous with matching pursuit algorithm. Before describing the computational method, let us to make a short mathematical description for scanned tissues.

The ultrasound imaging is a technique based on the physical properties of acoustical wave reflection when it finds an interface of two different regions with different densities along its propagation. This allows the consideration of the acquired signal as a collection of RF echoes with different amplitude size. Using the above presented considerations, one can say that the useful information, that is, the topological function of scanned tissues can be simulated as a sparse signal superimposed by white gaussian noise. Here, the high amplitude pulses simulate the strongest reflectors and correspond to edges/details of imaging target. The white gaussian noise will correspond to the speckle noise, which according to the final objective of ultrasound sequences processing must be eliminated, reduced, or preserved.

Using the sparsity constraint, we propose an algorithm which is able to reconstruct the original signal without inverting the PSF. This helps us to avoid the inverse problem, which has been known as one of the difficult problems in signal processing.

Within this approach it is considered that the problem could be divided into subproblems. Each subproblem has the objective to eliminate the influence of the most important reflector. In this way, it extracts iteratively from the envelope of the measured signal the influence of the most important blurred scatter; then replaces it with a unit pulse in the output signal, at the same position. At the beginning, it has all positions zero. The algorithm is a greedy algorithm since it works top-down. It provides a locally optimal choice to solve the subproblem, in the hope that at the end, the final solution is optimal [23]. For the implementation of the proposed deconvolution algorithm see Algorithm 1.

Here, *R*(*n*) is so called the residual signal, *R*_*i*_(*n*_*i*_) is the value of maximum amplitude at the position *n*_*i*_ at iteration *i*, and ⊗ is the convolution operator. For this study it was fixed that *k* = 0, because in this way the algorithm extracts the maximum number of possible reflectors.

Being an iterative deconvolution algorithm, its convergence must be studied. According to the condition of positiveness for the reflectivity function, the proposed algorithm iterations have sense, while the residual signal has values greater than zero. Also, the envelope of the PSF being a positive function, it results that the subtraction of a positive function from another positive one will generate a new residual function, at the iteration *i* + 1 which always satisfy the inequality *R*_*i*+1_(*n*) < *R*_*i*_(*n*). This condition is enough to prove that the algorithm will always reach the exit condition. The number of iterations corresponds with the sparsity coefficient, where sparsity coefficient means the number of nonzero elements in the final result. The PSF amplitude is normalized to preserve the same amplitude as in the envelope signal for the resulted sparse signal.

## 4. Results

The method was tested using synthetic RF-signals and real ultrasound sequences. The experiments with simulated signals are motivated by the allowance of quantitative evaluations under controlled conditions. Then the algorithms were applied to real data to test the feasibility of algorithms in clinical applications where the original topology of tissues is unknown. In the following, these two directions of evaluation will be presented as follows: Section 4.1 presents the results for simulated data and Section 4.2 shows the results for real data.

### 4.1. Experiments Using Simulated Signals

The so called *reflectivity function*, which simulates the tissues topology, was generated using Laplacian PDF assumption. For the simulations we generated sparse synthetic signals for reproduction of the strongest reflectors. It is contaminated with gaussian white noise to simulate the speckle noise. The length of the signals was 512 points, the sampling frequency was 20 MHz, and the central transducer frequency was 3.2 MHz. This corresponded to a sequence of 160 *μ*s and an approximately 3.94 cm deep scanning (for a standard ultrasound velocity *c* = 1540 m/s). During the experiments the above mentioned added gaussian white noise was generated according to different SNR values. With this noise we intended to simulate different types of tissues. For example, we find more speckle noise and weak scatters in the soft tissues, like abdominal tissues.

This reflectivity function must be transformed into an RF signal to simulate the acquired signal of the ultrasound probe. According to (1) it can be obtained if the reflectivity function is convolved with a simulated radio-frequency PSF. For current studies the RF PSF was generated using the formula [24]:

(9) $$\begin{array}{c} \text{PSF}=A\cdot \exp\left[-{\left(\frac{\omega t}{N\pi }\right)}^{2}\right]\sin\omega t, \end{array}$$

where *A* means the PSF amplitude, exp⁡ is the exponential function, *ω* is the angular frequency, *t* symbolizes time, and *N* is the number of the periods of the sinusoidal wave of the PSF. The use of this formula is motivated by its capability to control the number of oscillations in the simulated RF pulse. From the experiments it was observed that the sinusoidal wave had 3 or 4 periods.

**Figure 1:**
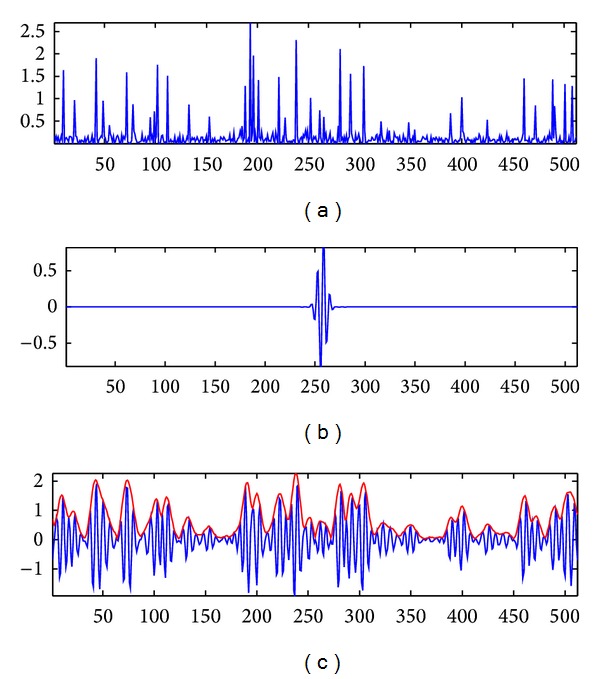
Simulated signals. (a) The generated reflectivity function. (b) The generated PSF. (c) The resulted RF signal and its envelope.

Figure 1 presents an RF simulated signal example as follows: Figure 1(a) represents simulated tissue reflectivity function, Figure 1(b) represents the generated RF PSF, and Figure 1(c) represents the RF obtained after convolution and its envelope.

Wavelet decomposition and denoising were performed using Wavelab Toolbox, downloaded from http://statweb.stanford.edu/~wavelab/. For the *σ* parameter in (7), it was observed experimentally that using 5 levels of decomposition was enough for a good elimination of the noise. Also, the estimation of the PSF was made under assumption of minimum phase.

The second step of the algorithm was the deconvolution. The current algorithm, described in Section 3.3 was compared also with different state of the art methods used in deconvolution as follows: regularized least square using *l*_1_-norm, Wiener filter (or *l*_2_-norm regularization), and total variation [3, 25].

The lagrangian parameter, *λ*, for comparative methods was fixed empirically to obtain the best results as follows: for *l*_1_-norm *λ* = 0.2, for Wiener filter (also known as Tikhonov regularization) *λ* = 0.08, and for TV-norm *λ* = 0.14.

The results were presented in terms of visual and quantitative evaluation. For quantitative measurements, we assessed the execution time for each method and we computed the normalized Mean Square Error (*n*MSE) and also resolution gain (RG) parameter. RG parameter is based on the ratio between normalized autocorrelation function of the original envelope and the resulted signal higher than −3 dB [12]. The *n*MSE is defined as follows:

(10) $$\begin{array}{c} n\text{MSE}=E\left[\frac{{||\hat{x}-x||}_{2}^{2}}{{||x||}_{2}^{2}}\right], \end{array}$$

where *E* is the statistical expectation, *x* is the original reflectivity function, and $\hat{x}$ is the resulted reflectivity function.

**Figure 2:**
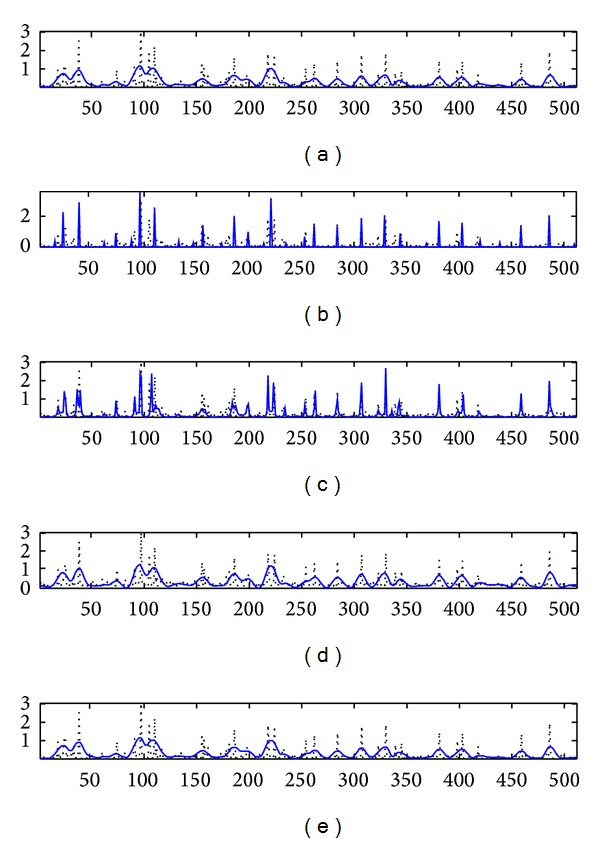
Simulated signals results. (a) Envelope of the simulated RF signal; (b) results obtained with our algorithm; (c) results obtained with *l*_1_-norm; (d) results obtained with Wiener filter; (e) results obtained with TV-norm. All signals are superimposed over original reflectivity function (dotted signal).

Figure 2 presents the results on simulated signals. It contains in Figure 2(a) the original RF signal envelope, which was used in all deconvolution methods as input signal; then the obtained results as follows: Figure 2(b): results were obtained with our algorithm, Figure 2(c): results obtained with *l*_1_-norm, Figure 2(d): results obtained with Wiener filter, and Figure 2(e): results obtained with TV-norm. For a better evaluation of the results, all signals were superimposed over the original reflectivity function (dotted signal). After computations, all results were normalized and then displayed to have the same dynamic ranges. It could be seen that our algorithm outperforms the comparative methods in terms of amplitude and scatters estimation. Almost all extracted peaks superimposed the original ones. *l*_1_-norm method offered also a sparse solution for the final result, but it could be seen that the final result was more contaminated with noise, which limits the approach for clinical investigations. The last two methods, Wiener filtering and TV-norm, offered smooth solutions which did not always offer well distinct or well contoured reflectors.

To complete the qualitative evaluation, the results were assessed using some numerical criteria.  Table 1 summarizes the results for the *n*MSE according to (10) and resolution gain. The displayed values are the results of trade off over 100 independently generated signals for all SNR values. In terms of *n*MSE it could be observed that the best results were offered by *l*_1_-norm followed closely by our method, but Wiener filter and TV-norm were outperformed and in terms of resolution gain the best results are offered by our method followed by *l*_1_-norm. Wiener filter and TV-norm had an insignificant resolution improvement.

Also, an important feature of the proposed algorithm was its execution time. In Table 2 it could be seen that our method outperformed all the compared techniques. This was the logical consequence of the fact that it worked directly in time domain and with the most important operation being vector subtraction. It must be mentioned that for execution time, deconvolution algorithms without PSF estimation were evaluated.

Table 3 showed the results of a statistical evaluation for scatters detection for proposed algorithms (our method, Wiener filter, and *l*_1_-norm). The evaluations were made using simulated signals using different levels of speckle noise and different number of scatters. It must be said that to make the same evaluation for Wiener filter result, we use a signal where we keep only all local maximums. The objective of this simulation was to evaluate the detection capability for each algorithm in different conditions. It could be observed that the proposed algorithm and *l*_1_-norm offer similar results and they have a bigger detection capacity than Wiener filter. This is normal because Wiener filters smooth the information and a part of small details was lost in the reconstruction process. From the point of view of real scatters discovery, we can observe that the more the number of scatters increased, the more the number of detected ones decreased. This can be explained if we refer to Rayleigh condition of superresolution. In the case of a high number of scatters a part of them cannot be recovered, the scatters that are closer to *λ*/2, where *λ* is the wavelength of the emitted PSF. This fact is visible also in Figure 2 (at the samples 250) where a part of them is not recovered.

### 4.2. Experiments on Real Ultrasound Sequences

As a next step, the proposed deconvolution algorithms were compared using real ultrasound sequences composing ultrasound images. As shown in synthetic signals evaluations, the ultrasound sequences can be done in the same procedure. The envelope could be obtained using Hilbert transform; then PSF was estimated for each sequence and finally the reflectivity function was estimated.

**Figure 3:**
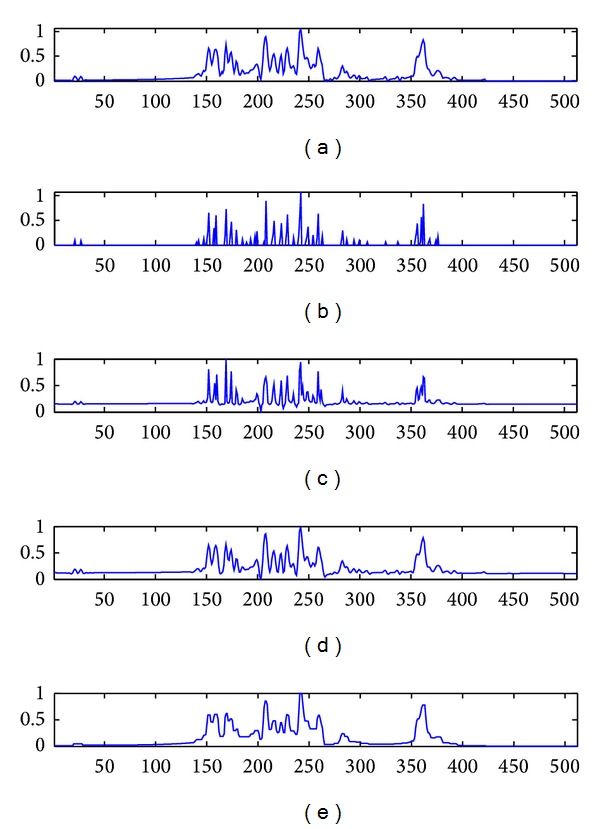
Measured signals results. (a) Envelope of measured signal; (b) results obtained with our algorithm; (c) results obtained with *l*_1_-norm; (d) results obtained with Wiener filter; (e) results obtained with TV-norm.

The first experiment on this section is focused on testing real independently measured signals. In the Figure 3 are shown the real measured signal in the subplot Figure 3(a) and then, the results of reconstruction for used algorithms, in the same order as in Figure 2. Because of no *a priori* information about the original reflectivity function it was impossible to evaluate the *n*MSE parameter and also to make the superimposition of the obtained results over the original reflectivity function. For that, the qualitative evaluation is completed using just the RG parameter. In Table 4 the results of quantitative evaluation for the evaluated methods using this parameter are presented. Both visual and quantitative evaluations validated the results obtained for the synthetic signals. It can be seen that our method outperforms the Wiener filter and TV-norm and offers similar results with *l*_1_-norm.

**Figure 4:**
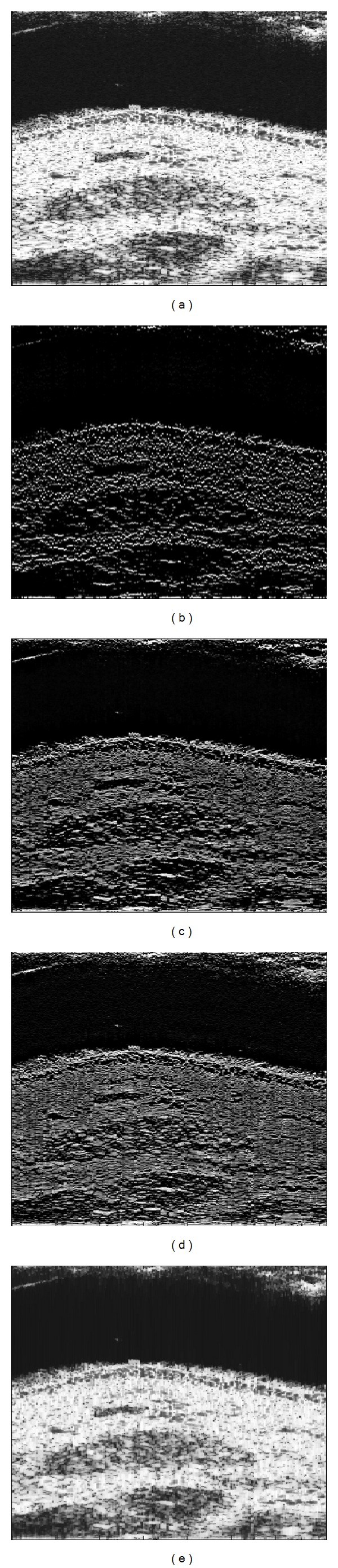
(a) Original data; (b) proposed method; (c) *l*_1_-norm method; (d) Wiener filtering; and (e) TV-norm method.

Then, in our experiments we used multiple images obtained in our laboratory.  Figure 4 is a log-compressed B-mode image of the skin obtained by an ultrasound scanner developed in-house called Ecoderm. The probe used with this imaging device is a 128 elements linear array working at 20 MHz center frequency with 87% relative bandwidth. The linear scan is performed by the scanner through an emission aperture composed of 15 elements having focalization delays set up for 8 mm in soft tissues. For computation constraint all the sequences were zero-padded until the next 2^power^ value.

In terms of visual evaluation our method outperformed the comparative techniques. Here, the reflectors are more visible and the speckle noise, which reduces the image quality, was almost suppressed. Moreover, the contours were more visible and the regions without reflectors were better distinguished.

The proposed algorithm assumes that the signal to be recovered is sparse; that is, it has a Laplacian distribution. For that it and *l*_1_-norm are more adapted to reconstruct ultrasound images of tissues with a small number of scatters. This means that in the final result the important details are furthermore revealed and the smaller details (i.e., the speckle noise) are reduced or eliminated. Such similar behavior is observed also in the synthetic signals and real sequences.

As expected, in some cases, the sparse reflectivity sequence is difficult to be interpreted directly because of speckle noise suppression. Some possible improvement can be made for a more realistic interpretation like convolution with an ideal PSF or superimposition of the sparse data over B-mode image.

## 5. Discussion and Conclusion

The present paper addresses the problem of blind deconvolution for ultrasound sequences in medical imaging by formulating a solution that is able to extract the reflectivity function avoiding the hard problem of inverse filtering. The proposed algorithm is a time domain blind deconvolution that works as a greedy algorithm. The solution estimates in a blind way the PSF, and then, it extracts iteratively the tissue reflectivity function using the estimated PSF.

Being a blind technique, it was assumed *a priori* that the reflectivity function had a sparse shape (i.e., it follows the Laplacian distribution law). Another important feature of this method is its execution time. From the accomplished experiments, it can be seen that the greedy algorithm method outperforms the most used methods in the domain. Also the algorithm works using the envelope of the acquired RF signals, which avoid the problem of the acoustic wave central frequency estimation.

It is well known that in its moving along the propagation direction the PSF shape is changing according to attenuation/nonlinear effects in the tissues. Generally, for perfect results the state of the art approaches divide the image in sections and then the PSF is calculated locally for each section. In reconstruction, for each section, it is used the locally estimated PSF with the same deconvolution algorithm. This means that the deconvolution algorithms work identically, but the results change because of the different used PSFs. The purpose of this research is to prove their feasibility for ultrasound sequences; therefore we only considered the nonvariant case in the experimentations.

From the simulations it resulted as well that scatters were well identified and the speckle noise was almost suppressed. However, in some conditions the results were too sparse and this could create some difficulties in information interpretation.

Finally, a number of future works can be outlined. First, the next step will be to analyze the proposed method for different types of tissues in a clinical investigation. Also, we can try to improve the algorithm by imposing supplementary constraints for a better interfaces detection in the situations when they are very close. As discussed before, the sparsity constraint is not always well suitable and this could be improved by making a convolution of the resulted sparse signal with a PSF as in [26]. Choosing the width for PSF can be an interesting study and can offer different solutions according to desired application.

## Figures and Tables

**Algorithm 1:**
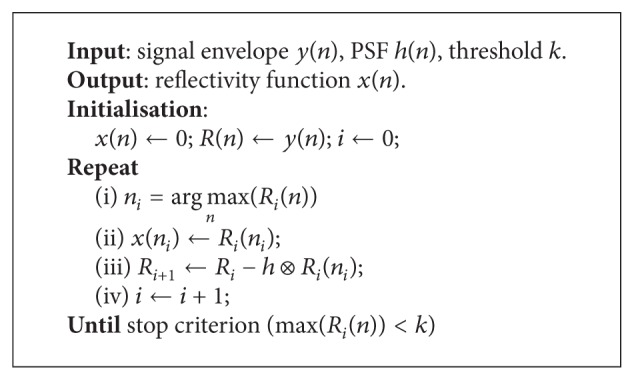
Noninverse greedy deconvolution.

**Table 1:** Comparison of different restoration techniques according to *n*MSE (*n*-Mean Square Error) from (10) and RG (resolution gain). RG is a parameter which evaluates the level of decorrelation for speckle noise in the resulted signal.

Methods	SNR = 7 dB		SNR = 14 dB		SNR = 21 dB	
	*n*MSE	RG	*n*MSE	RG	*n*MSE	RG
Our method	1.36	17.56	1.17	15.48	1.05	14.28
*l*_1_-norm	1.18	17.04	1.11	15.02	0.98	13.82
Wiener	2.82	2.15	2.62	1.68	2.69	1.52
TV-norm	2.52	0.76	2.33	0.87	2.32	0.91

**Table 2:** Execution time evaluation for tested algorithms.

	Our alg.	*l*_1_-norm	Wiener	TV-norm
Time (s)	0.002	19.01	0.7	3.83

**Table 3:** Real scatters detection according to their density.

Density	Method	SNR (dB)				
		5	10	15	20	25
2%	Our. alg.	9.05	9.07	9.07	9.12	9.15
	Wiener	8.38	8.76	8.68	8.68	8.86
	*l*_1_-norm	8.94	8.95	8.92	8.96	8.94

5%	Our. alg.	18.9	19.00	19.68	19.72	20.06
	Wiener	18.48	19.56	19.62	19.78	19.88
	*l*_1_-norm	18.6	19.06	19.70	19.74	19.98

10%	Our. alg.	37.62	37.94	37.62	38.30	39.02
	Wiener	34.34	34.62	34.74	34.8	35.16
	*l*_1_-norm	38.62	39.50	39.80	39.56	39.92

**Table 4:** Resolution gain evaluation on measured signals.

Criteria	Our alg.	*l*_1_-norm	Wiener	TV-norm
RG	15	15	3	1

## References

[1] Szabo TL (2004). *Diagnostic Ultrasound Imaging: Inside Out*.

[2] Park SC, Park MK, Kang MG (2003). Super-resolution image reconstruction: a technical overview. *IEEE Signal Processing Magazine*.

[3] Campisi P, Egiazarian K (2007). *Blind Image Deconvolution: Theory and Applications*.

[4] Hundt EE, Trautenberg EA (1980). Digital processing of ultrasonic data by deconvolution. *IEEE Transactions on Sonics and Ultrasonics*.

[5] Abeyratne UR, Petropulu AP, Reid JM (1995). Higher order spectra based deconvolution of ultrasound images. *IEEE Transactions on Ultrasonics, Ferroelectrics, and Frequency Control*.

[6] Taxt T, Strand J (2001). Two-dimensional dimensional blind deconvolution of ultra-sound images. *IEEE Transactions on Ultrasonics, Ferroelectrics, and Frequency Control*.

[7] Michailovich OV, Adam D (2005). A novel approach to the 2-D blind deconvolution problem in medical ultrasound. *IEEE Transactions on Medical Imaging*.

[8] Ng J, Prager R, Kingsbury N, Treece G, Gee A (2007). Wavelet restoration of medical pulse-echo ultrasound images in an em framework. *IEEE Transactions on Ultrasonics, Ferroelectrics, and Frequency Control*.

[9] Michailovich O, Tannenbaum A (2007). Blind deconvolution of medical ultrasound images: a parametric inverse filtering approach. *IEEE Transactions on Image Processing*.

[10] Yu C, Zhang C, Xie L (2012). An envelope signal based deconvolution algorithm for ultrasound imaging. *Signal Processing*.

[11] Vollmann W (1982). Resolution enhancement of ultrasonic B-scan images by deconvolution. *IEEE Transactions on Sonics and Ultrasonics*.

[12] Taxt T (1995). Restoration of medical ultrasound images using two-dimensional homomorphic deconvolution. *IEEE Transactions on Ultrasonics, Ferroelectrics, and Frequency Control*.

[13] Strand J, Taxt T, Jain AK (1999). Two-dimensional phase unwrapping using a block least-squares method. *IEEE Transactions on Image Processing*.

[14] Taxt T (2001). Three-dimensional blind deconvolution of ultrasound images. *IEEE Transactions on Ultrasonics, Ferroelectrics, and Frequency Control*.

[15] Michailovich O, Adam D (2003). Robust estimation of ultrasound pulses using outlier-resistant de-noising. *IEEE Transactions on Medical Imaging*.

[16] Donoho DL (1995). De-noising by soft-thresholding. *IEEE Transactions on Information Theory*.

[17] Bruce AG, Donoho DL, Gao HY, Martin RD Denoising and robust nonlinear wavelet analysis.

[18] Taxt T, Strand J (2001). Two-dimensional noise-robust blind deconvolution of ultrasound images. *IEEE Transactions on Ultrasonics, Ferroelectrics, and Frequency Control*.

[19] Mallat SG, Zhang Z (1993). Matching pursuits with time-frequency dictionaries. *IEEE Transactions on Signal Processing*.

[20] Bertero E, Boccacci P (1998). *Introduction To Inverse Problem in Imaging*.

[21] Oppenheim AV, Schafer RW (1989). *Discrete Time Signal Processing*.

[22] Mallat S (2009). *A Wavelet Tour of Signal Processing: The Sparse Way*.

[23] Cormen TH, Leiserson CE, Rivest RL, Stein C (2009). *Introduction to Algorithms*.

[24] Bouakaz A, Palanchon P, Jong N (2007). Dynamique de la microbulle. *Échographie de contraste*.

[25] Rudin LI, Osher S, Fatemi E (1992). Nonlinear total variation based noise removal algorithms. *Physica D*.

[26] Högbom JA (1974). Aperture synthesis with a non-regular distribution of interferom baselines. *Astronomy and Astrophysics Supplement*.

